# Supplementary figures and images for: Dystonin modifiers of junctional epidermolysis bullosa and models of epidermolysis bullosa simplex without dystonia musculorum
Source: PLoS One. 2023 Oct 26;18(10):e0293218. doi: 10.1371/journal.pone.0293218 (PMC10602294; doi:10.1371/journal.pone.0293218)

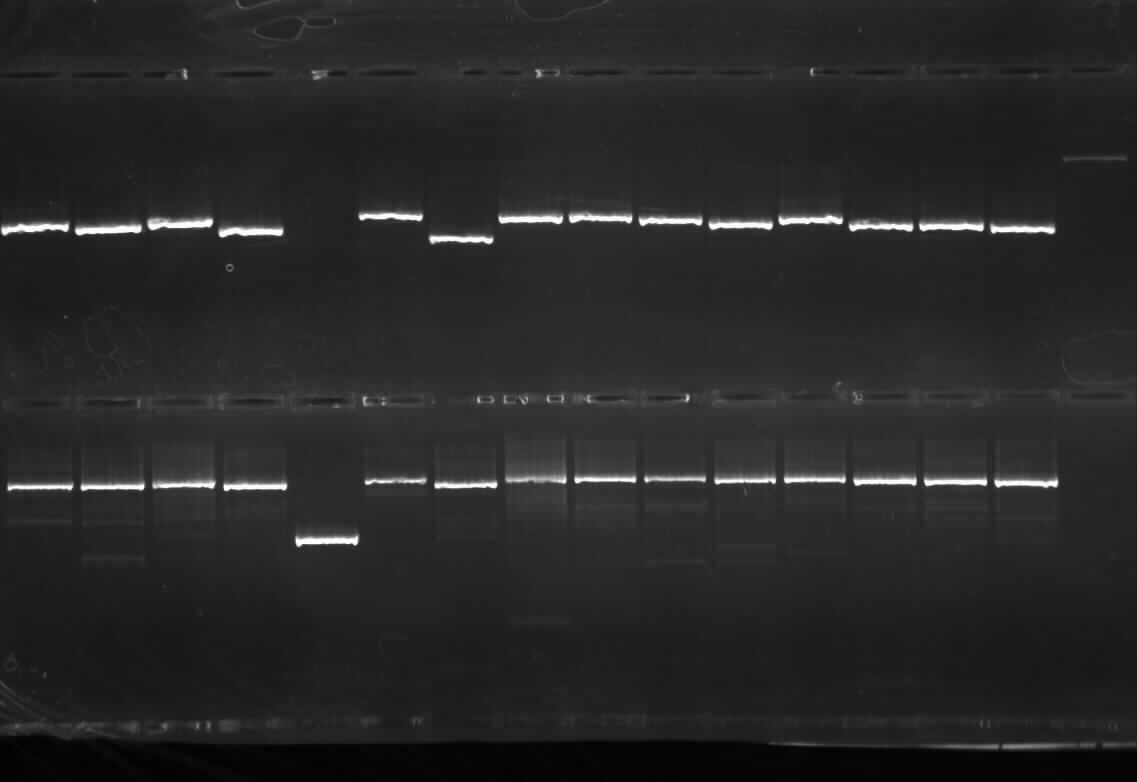

Supplement: S1 Fig — (JPG) [file pone.0293218.s002.jpg]

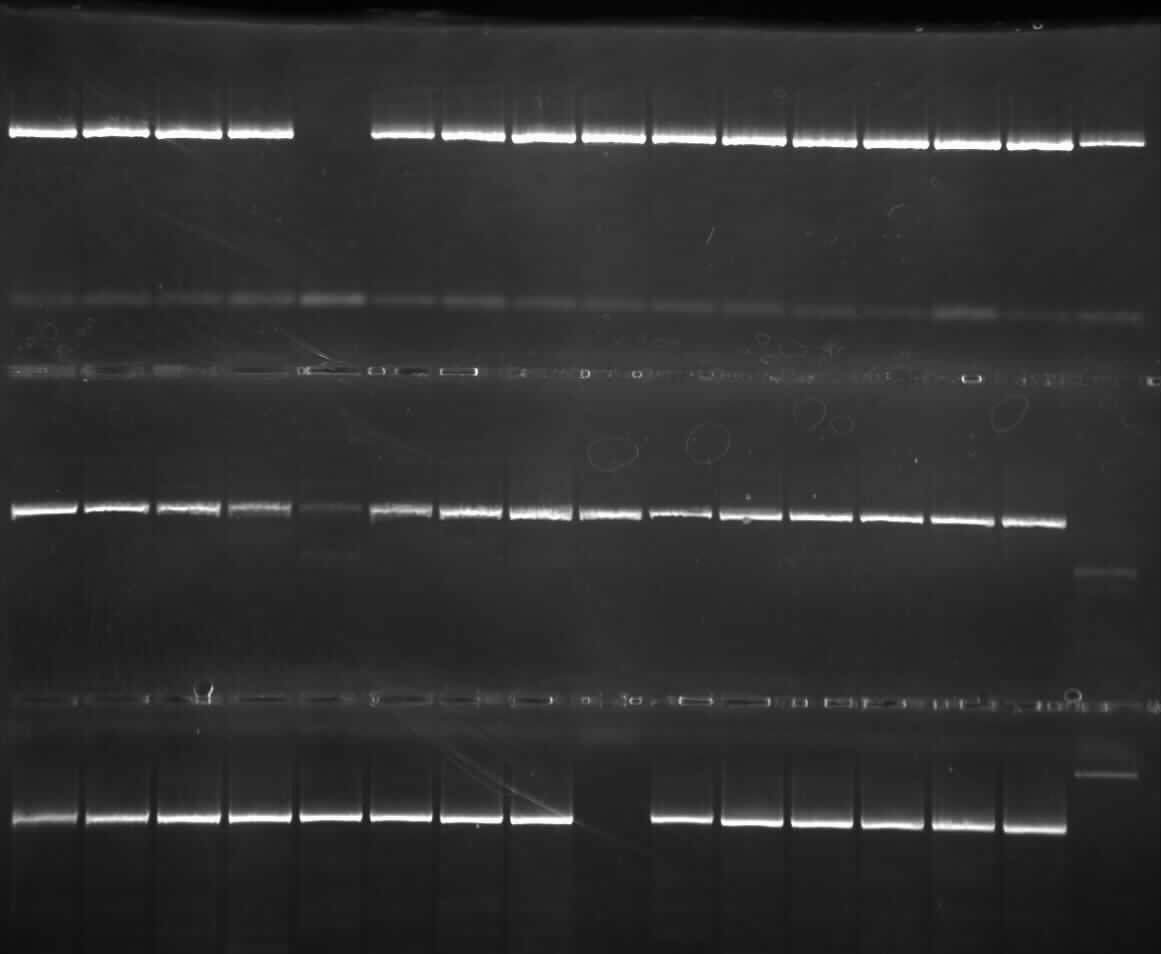

Supplement: S2 Fig — (JPG) [file pone.0293218.s003.jpg]

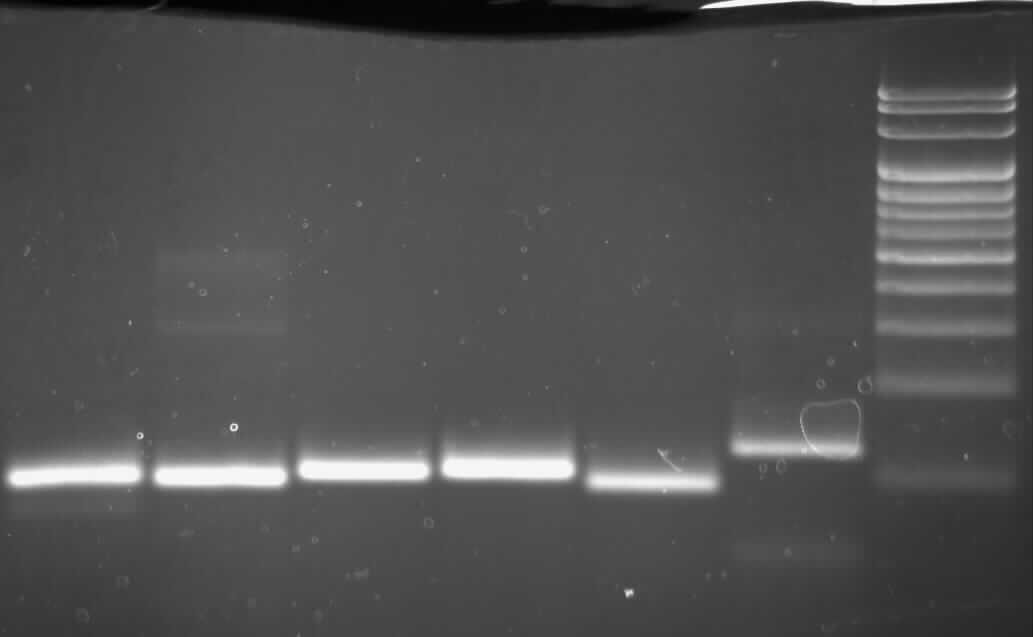

Supplement: S3 Fig — (JPG) [file pone.0293218.s004.jpg]
