# Supplementary material for: Dystonin modifiers of junctional epidermolysis bullosa and models of epidermolysis bullosa simplex without dystonia musculorum
Source: PLoS One. 2023 Oct 26;18(10):e0293218. doi: 10.1371/journal.pone.0293218 (PMC10602294; doi:10.1371/journal.pone.0293218)
Supplement: S1 File — (PDF) [file pone.0293218.s005.pdf]

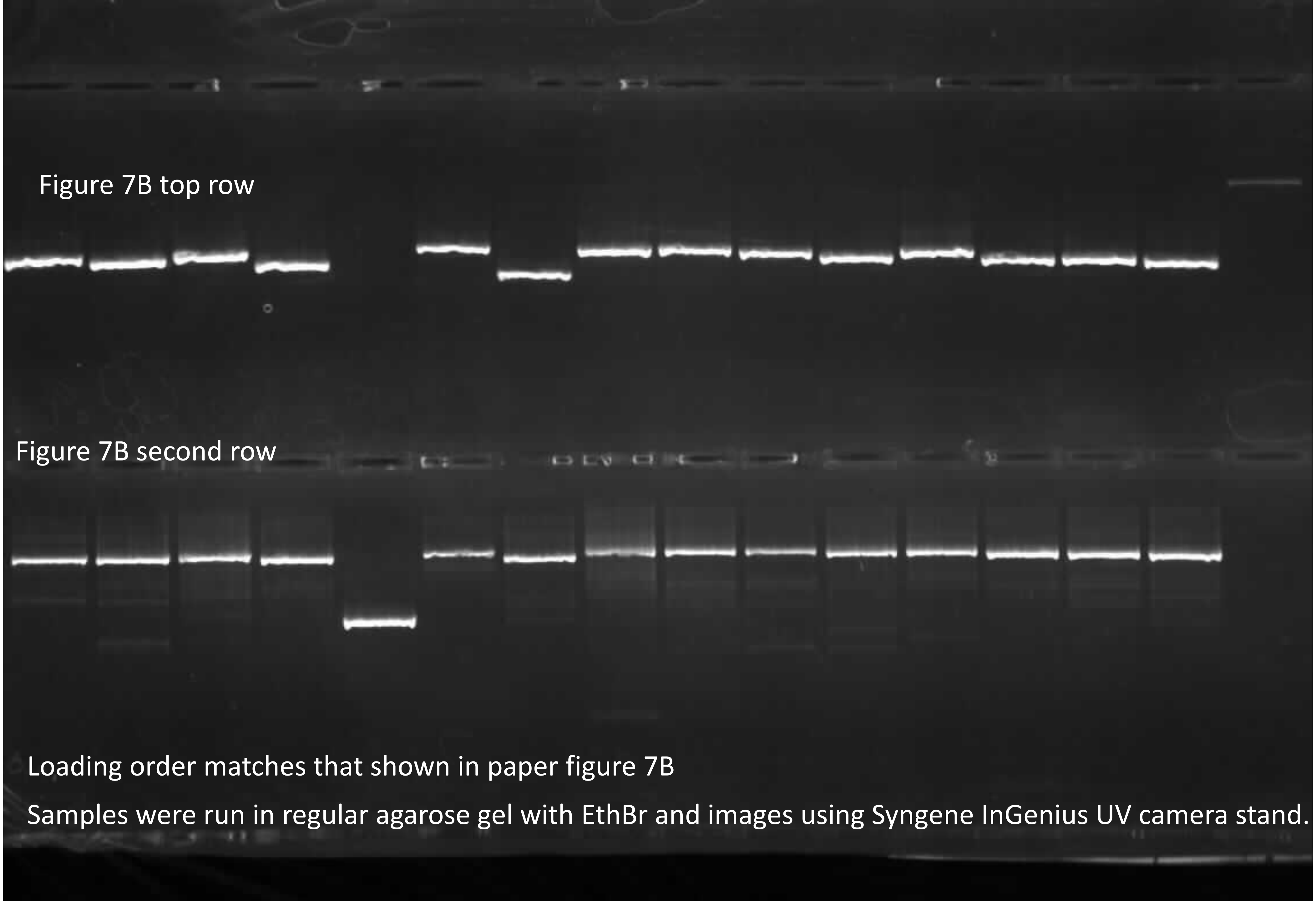

Loading order matches that shown in paper figure 7B

Samples were run in regular agarose gel with EthBr and images using Syngene InGenius UV camera stand.

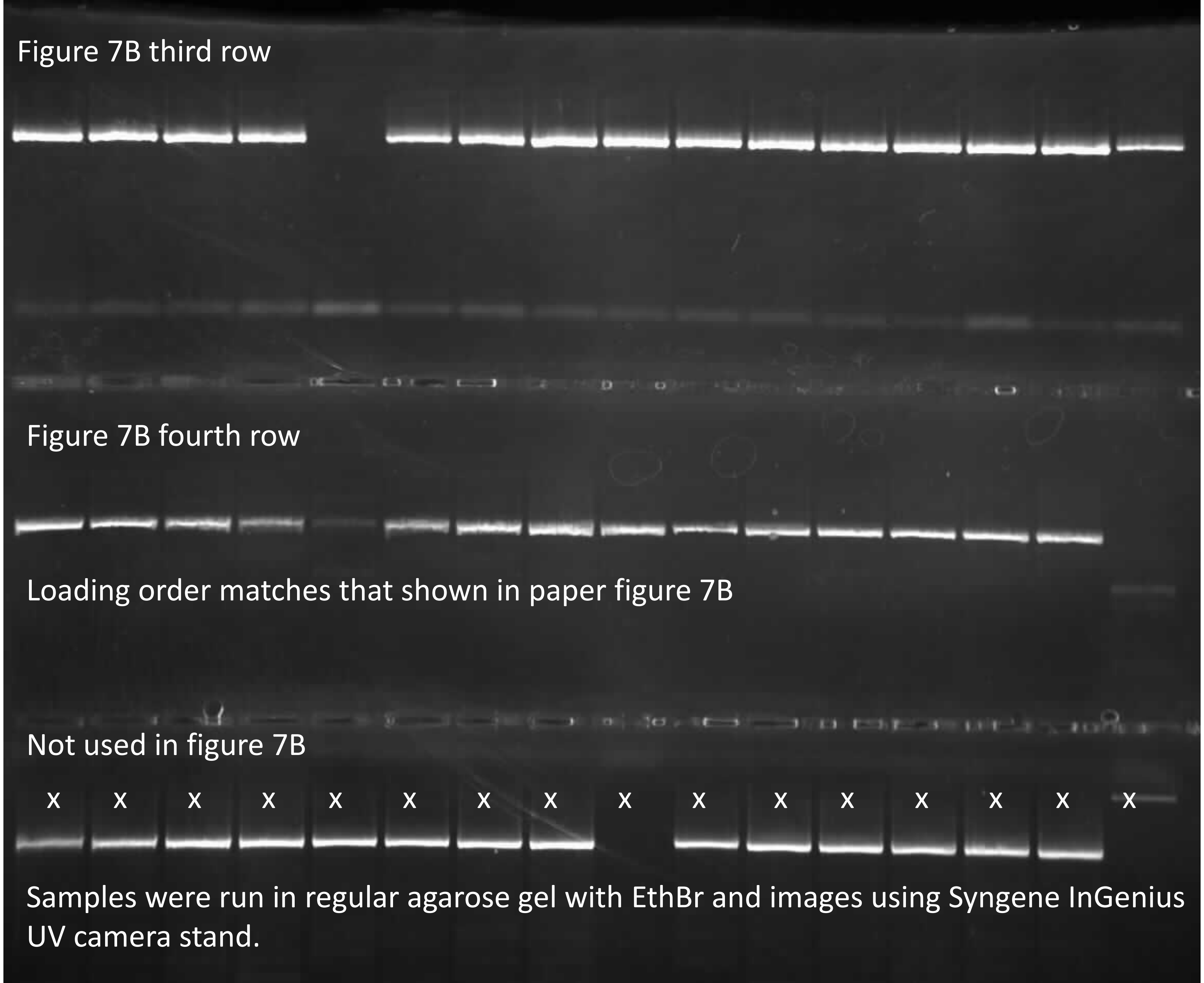
Figure 7B third row
Figure 7B fourth row
Loading order matches that shown in paper figure 7B
Not used in figure 7B
x x x x x x x x x x x x x x x x
Samples were run in regular agarose gel with EthBr and images using Syngene InGenius UV camera stand.

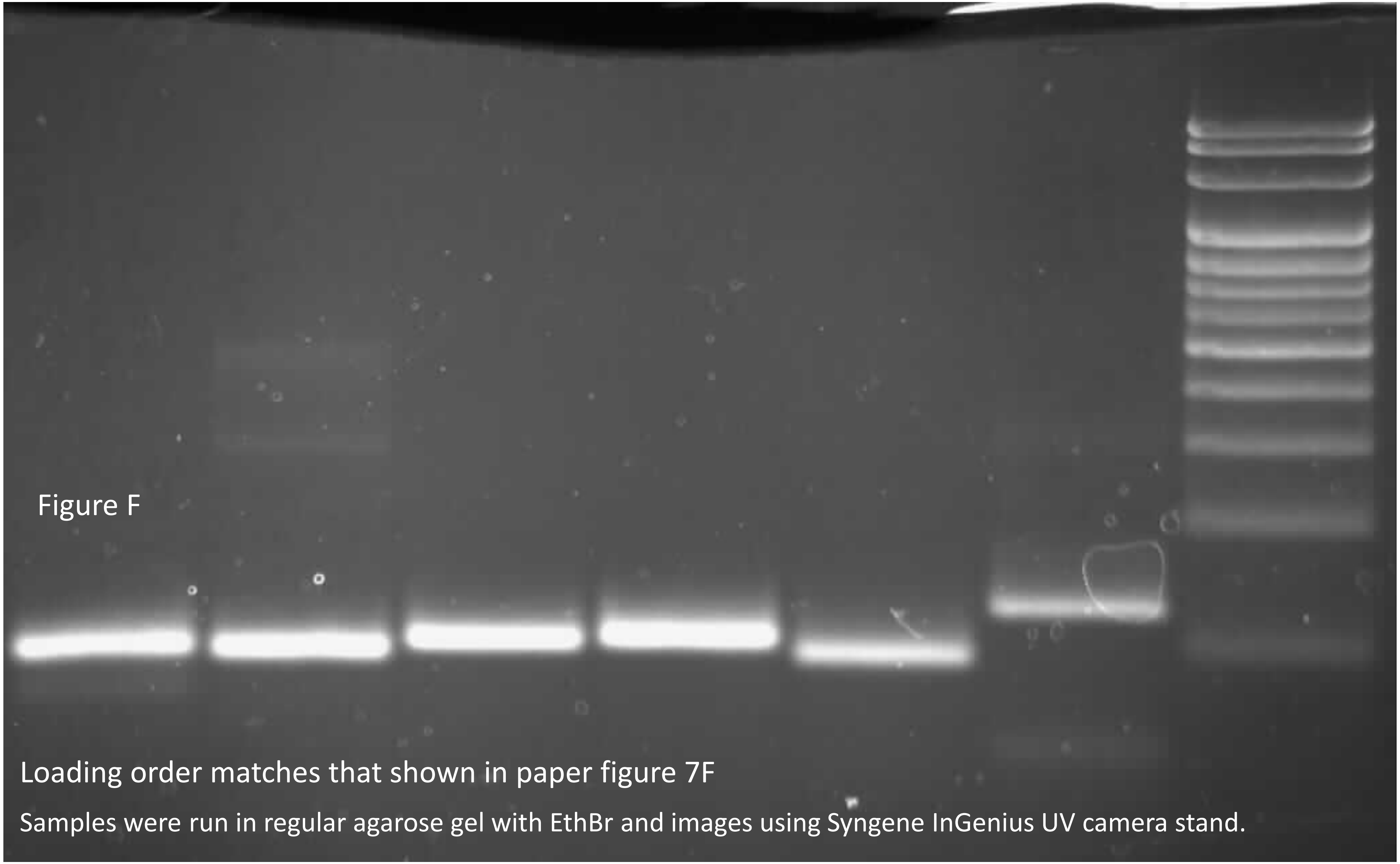
Figure F
Loading order matches that shown in paper figure 7F
Samples were run in regular agarose gel with EthBr and images using Syngene InGenius UV camera stand.
