# Supplementary material for: Dystonin modifiers of junctional epidermolysis bullosa and models of epidermolysis bullosa simplex without dystonia musculorum
Source: PLoS One. 2023 Oct 26;18(10):e0293218. doi: 10.1371/journal.pone.0293218 (PMC10602294; doi:10.1371/journal.pone.0293218)
Supplement: Attachment — Submitted filename: Response to Reviewers.docx [file pone.0293218.s006.docx]

Response to Reviewers

Thank you for your thoughtful comments concerning our submitted manuscript PONE-D-23-22545 *Dystonin modifiers of junctional epidermolysis bullosa and models of epidermolysis bullosa simplex without dystonia musculorum*.

Editor comments.

“Although neither one of the reviewers suggested additional experiments, reviewer 2 felt - and I agree - that the manuscript could benefit from streamlining including shortening of the text and restructuring of data presentation. Several additional points raised need clarification and consideration.”

Response: We have made another pass through the manuscript resulting in some clarification and modest streamlining.

Reviewer 1 comments.

“In some figures, B6 is used to represent *Lamc2^wt/wt^*, but in others, B6 stands for *Lamc2^jeb/jeb^*. Consistency in nomenclature across all figures would be preferable.”

Response: Figures 3 and 10 have been changed to have B6-jeb stand for *Lamc2^jeb/jeb^*.

“The rediscovery of the rodless *Dst1* isoform is intriguing. Please emphasize that the removal of exon 23 is in-frame, enabling readers to more readily understand that exon 23 skipping does not lead to nonsense-mediated mRNA decay.”

Response: Changes were made to emphasize this.

“Is it possible that pinnae removal could improve inflammatory phenotypes observed in other EB model mice (e.g., *Lama3* targeted disruption; PMID 27729280)? If so, please make a note of it in the manuscript.”

Response: We added a sentence to the discussion suggesting it may be worth exploring pinnae removal in other mouse EB models.

Reviewer 2 comments.

“In the introduction it is stated in regard to modifiers of disease in epidermolysis bullosa (lines 46-48) that no additional modifiers apart from MMP1 have been identified. This statement is in my opinion too strong. There has been associations in patients made of higher decorin expression and milder diseases in recessive dystrophic epidermolysis bullosa (Odorisio et al., Human Mol Genet 2014), a similar link was made for PRELP (Chacon-Solano et al., Matrix Biol 2022).”

Response: This statement has been modified and both of the citations mentioned here added to references.

“The correct abbreviation for laminin-332 is LM332.”

Response: Actually this statement is not correct. We checked with Michelle Perry, PhD, of Mouse Genome Informatics at The Jackson Laboratory. She is the contact person for all questions on mouse genetic nomenclature. She stated “We don’t have official nomenclature guidelines for protein complexes. The more common version (at least from Google hits) appears to be Ln332 rather than L332 or LM332.” Therefore, we decided to leave it as L332 to match our previous publication.

“The observations of pinnae removal delaying development of severe phenotypes and extending healthy span are interesting. The suggestion that is secondary due to reduced scratching of ears is reasonable. However, did the authors also consider autoimmunity?”

Response: Autoimmunity is one of many interesting possible explanations. The lab has closed down and we no longer have the option to test any of these. We only mention scratching as a ‘most likely’ possible explanation.

“The discussion is interesting but I think it is too long to be effective. I would recommend to shorten it an focus on discussing essential findings.”

Response: We have made efforts to clarify and condense where we could, resulting in modest shortening of the text. We hope this is sufficient to satisfy the reviewer and editor.
